# Supplementary material for: Effects of Starch Overload and Cecal Buffering on Fecal Microbiota of Horses
Source: Animals (Basel). 2022 Dec 6;12(23):3435. doi: 10.3390/ani12233435 (PMC9737938; doi:10.3390/ani12233435)

Figure S1. Production and characteristics of horses' feces before and after starch overload. (A) before overload - feces in cymbals and normal consistency; (B) 12 to 24 hours after overload - pasty feces with white color; (C) 48 hours after overload - feces returning to cymbals and normal consistency.

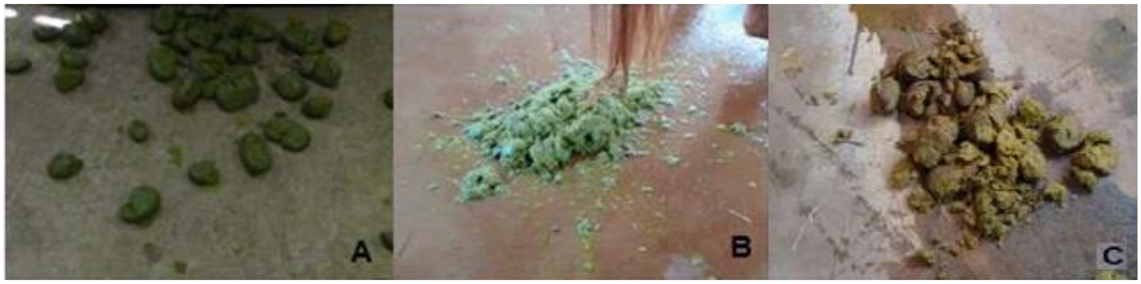

Supplement: Supplementary file 1 [file animals-12-03435-s001.zip › Figure S1.pdf]
