# Supplementary material for: Effects of Starch Overload and Cecal Buffering on Fecal Microbiota of Horses
Source: Animals (Basel). 2022 Dec 6;12(23):3435. doi: 10.3390/ani12233435 (PMC9737938; doi:10.3390/ani12233435)
Supplement: Supplementary file 1 [file animals-12-03435-s001.zip › Table S1.pdf]

Table S1. Parameters evaluated (movement, signs of abdominal discomfort, feces production, food intake, rectal temperature, heart rate) for each animal from starch-saline and starch-buffer treatments, during the experimental period.

	SS							SB					
	**T0**	**T8**	**T12**	**T24**	**T48**	**T72**		**T0**	**T8**	**T12**	**T24**	**T48**	**T72**
**ANIMAL 1**							**ANIMAL 6**						
**HR**	40	60	60	52	40	36		32	42	50	52	76	68
**T°C**	37.1	37.5	37.8	37.2	37.4	37.8		36.5	37.5	39.3	39.1	38.4	37.0
**Diarrhea**	-	-	-	YES	-	-		-	-	YES	YES	YES	-
**Hyporexia**	-	-	-	-	-	-		-	-	-	YES	YES	YES
**Decubitus**	-	-	-	-	-	-		-	-	-	-	YES	YES
**Dehydration**	-	-	-	-	-	-		-	-	5%	10%	10%	5%
**N° steps**	8	8	8	8	10	8		8	8	9	9	11	12
**Obel Grade**	-	-	-	-	-	-		-	-	-	-	2	4
**ANIMAL 2**							**ANIMAL 7**						
**HR**	36	56	40	40	44	80		36	44	62	48	80	68
**T°C**	37.1	37.9	37.6	37.1	38.4	38.5		36.4	38.7	39.2	38.0	38.6	39.3
**Diarrhea**	-	-	-	YES	-	-		-	-	YES	YES	YES	-
**Hyporexia**	-	-	-	-	-	-		-	-	-	YES	YES	-
**Decubitus**	-	-	-	-	-	-		-	-	YES	-	-	-
**Dehydration**	-	-	-	-	-	-		-	-	5%	10%	10%	-
**N° steps**	8	8	8	8	10	10		8	9	9	10	14	12
**Obel Grade**	-	-	-	-	2	3		-	-	-	-	2	3
**ANIMAL 3**							**ANIMAL 8**						
**HR**	40	56	56	56	52	64		50	50	62	60	60	44
**T°C**	37.4	37.6	37.8	38.0	37.6	37.3		37.9	38.4	39.9	38.7	38.2	36.7
**Diarrhea**	-	-	-	YES	-	-		-	-	-	YES	-	-
**Hyporexia**	-	-	-	-	-	-		-	YES	YES	YES	YES	-
**Decubitus**	-	-	-	-	-	-		-	-	-	-	-	-
**Dehydration**	-	-	-	-	-	-		-	-	-	5%	-	-
**N° steps**	8	8	8	8	8	8		9	9	9	9	12	10
**Obel Grade**	-	-	-	-	-	-		-	-	-	-	2	-
**ANIMAL 4**							**ANIMAL 9**						
**HR**	44	60	56	56	60	64		46	42	44	58	88	72

**T°C**	37.4	37.6	37.2	37.4	37.7	37.5		37.4	37.4	38.0	36.9	378	37.2
**Diarrhea**	-	-	-	YES	-	-		-	-	-	YES	YES	-
**Hyporexia**	-	-	-	-	-	-		-	YES	YES	YES	YES	YES
**Decubitus**	-	-	-	-	-	-		-	YES	-	-	-	YES
**Dehydration**	-	-	-	-	-	-		-	-	-	5%	5%	10%
**N° steps**	8	8	8	8	8	8		7	9	9	9	12	10
**Obel Grade**	-	-	-	-	-	-		-	-	-	-	3	3
**ANIMAL 5**							**ANIMAL 10**						
**HR**	36	56	60	48	48	52		36	44	74	52	44	44
**T°C**	37.2	37.9	38.2	37.5	37.4	37.4		38.1	38.8	19.9	38.5	37.5	37.5
**Diarrhea**	-	-	-	YES	-	-		-	-	YES	YES	-	-
**Hyporexia**	-	-	-	-	-	-		-	-	-	-	YES	-
**Decubitus**	-	-	-	-	-	-		-	YES	-	-	-	-
**Dehydration**	-	-	-	-	-	-		-	5%	5%	5%	-	-
**N° steps**	8	8	8	8	9	10		8	8	8	9	10	8
**Obel Grade**	-	-	-	-	2	2		-	-	-	-	-	-

SS = starch-saline; SB = starch-buffer; HR = heart rate (beats/minute); T°C = rectal temperature; N° steps = number of steps. T0 – before administration (starch overload and/or buffer); T8 – 8 hours administration; T12 – 12 hours after administration; T24 – 24 hours after administration; T48 – 48 hours after administration; T72 – 72 hours after administration.
