# Supplementary material for: Effects of Starch Overload and Cecal Buffering on Fecal Microbiota of Horses
Source: Animals (Basel). 2022 Dec 6;12(23):3435. doi: 10.3390/ani12233435 (PMC9737938; doi:10.3390/ani12233435)
Supplement: Supplementary file 1 [file animals-12-03435-s001.zip › Tables S2--S4.pdf]

Table S2. Variation in the relative abundance of the bacterial phyla in the feces of horses 72 hours following treatments.

| **Phylum** | **Treatments** | **Time** | | | | | | **Time** | **T*T** |
|---|---|---|---|---|---|---|---|---|---|
| | | T0 | T8 | T12 | T24 | T48 | T72 | P value | |
| Actinobacteria | | | | | | | | | |
| | WS | 0.011 | 0.009 | 0.009 | 0.006 | 0.008 | 0.006$^{B}$ | **.014** | **.011** |
| | WB | 0.010 | 0.005 | 0.004 | 0.008 | 0.004 | 0.008$^{B}$ | | |
| | SS | 0.012 | 0.009 | 0.007 | 0.013 | 0.055 | 0.023$^{B}$ | | |
| | SB | 0.008$^{b}$ | 0.005$^{b}$ | 0.004$^{b}$ | 0.033$^{b}$ | 0.156$^{ab}$ | 0.201$^{Aa}$ | | |
| Bacteroidetes | | | | | | | | | |
| | WS | 0.018 | 0.016 | 0.012 | 0.019 | 0.014$^{B}$ | 0.026$^{B}$ | **.013** | **<.001** |
| | WB | 0.019 | 0.027 | 0.020 | 0.019 | 0.035$^{B}$ | 0.028$^{B}$ | | |
| | SS | 0.017 | 0.023 | 0.026 | 0.068 | 0.025$^{B}$ | 0.025$^{B}$ | | |
| | SB | 0.021$^{b}$ | 0.030$^{b}$ | 0.002$^{b}$ | 0.001$^{b}$ | 0.218$^{Aa}$ | 0.184$^{Aa}$ | | |
| Euryarcheota | | | | | | | | | |
| | WS | 0.054 | 0.040 | 0.061$^{A}$ | 0.022 | 0.021 | 0.016$^{A}$ | **.001** | **.006** |
| | WB | 0.050 | 0.020 | 0.030$^{A}$ | 0.039 | 0.001 | 0.040$^{A}$ | | |
| | SS | 0.036 | 0.015 | 0.019$^{A}$ | 0.011 | 0.012 | 0.049$^{A}$ | | |
| | SB | 0.035$^{a}$ | 0.030$^{a}$ | 0.134$^{Bc}$ | 0.025$^{a}$ | 0.003$^{b}$ | <0.001$^{Bb}$ | | |
| Firmicutes | | | | | | | | | |
| | WS | 0.446 | 0.443 | 0.354$^{B}$ | 0.397$^{B}$ | 0.376 | 0.342 | **<.001** | **<.001** |
| | WB | 0.333 | 0.288 | 0.288$^{B}$ | 0.348$^{B}$ | 0.347 | 0.339 | | |
| | SS | 0.306 | 0.325 | 0.363$^{B}$ | 0.440$^{B}$ | 0.511 | 0.364 | | |
| | SB | 0.362$^{c}$ | 0.330$^{c}$ | 0.732$^{Aab}$ | 0.906$^{Aa}$ | 0.592$^{bc}$ | 0.530$^{bc}$ | | |
| Proteobacteria | | | | | | | | | |
| | WS | 0.004 | 0.003 | 0.001 | 0.006$^{B}$ | 0.002 | 0.003 | **.006** | **.050** |
| | WB | 0.009 | 0.008 | 0.009 | 0.006$^{B}$ | 0.002 | 0.008 | | |
| | SS | 0.010$^{b}$ | 0.006$^{b}$ | 0.009$^{b}$ | 0.059$^{Aa}$ | 0.018$^{ab}$ | 0.009$^{b}$ | | |
| | SB | 0.002 | 0.002 | 0.001 | 0.002$^{B}$ | 0.008 | 0.004 | | |
| Spirochaetes | | | | | | | | | |
| | WS | 0.029 | 0.030 | 0.035 | 0.028 | 0.027 | 0.040$^{A}$ | **.002** | **<.001** |
| | WB | 0.022 | 0.028 | 0.024 | 0.018 | 0.039 | 0.030$^{A}$ | | |
| | SS | 0.026 | 0.024 | 0.018 | 0.011 | 0.017 | 0.011$^{A}$ | | |
| | SB | 0.017$^{a}$ | 0.047$^{a}$ | 0.006$^{b}$ | 0.002$^{b}$ | 0.001$^{b}$ | <0.001$^{Bb}$ | | |

WS = water-saline; WB = water-buffer; SS = starch-saline; SB = starch-buffer. T*T = interaction treatment/time. T0 – before gastric administration of water or starch; T8 - 08h; T12 - 12h; T24 - 24h; T48 - 48h; T72 - 72h. The means are represented: different lowercase letters on the same line, and different uppercase letters in the same column differed statistically (*p<0.05*; Tukey's test).

Table S3. Variation in the relative abundance of the bacterial families in the feces of horses 72 hours following treatments.

| Family | Treatment | Time | | | | | | Time | T*T |
|---|---|---|---|---|---|---|---|---|---|
| | | T0 | T8 | T12 | T24 | T48 | T72 | P value | |
| Bifidobacteriaceae | | | | | | | | | |
| | WS | <0.001 | <0.001 | <0.001 | <0.001 | <0.001$^{B}$ | <0.001$^{B}$ | **.015** | **.020** |
| | WB | <0.001 | <0.001 | <0.001 | <0.001 | <0.001$^{B}$ | <0.001$^{B}$ | | |
| | SS | <0.001 | <0.001 | <0.001 | 0.007 | 0.050$^{B}$ | 0.015$^{B}$ | | |
| | SB | <0.001$^{b}$ | <0.001$^{b}$ | <0.001$^{b}$ | 0.031$^{b}$ | 0.146$^{Aa}$ | 0.195$^{Aa}$ | | |
| Erysipelothricaceae | | | | | | | | | |
| | WS | <0.001 | 0.001 | 0.001 | 0.001 | 0.001$^{B}$ | 0.001$^{B}$ | **.001** | **<.001** |
| | WB | 0.002 | 0.002 | 0.003 | 0.003 | 0.001$^{B}$ | 0.002$^{B}$ | | |
| | SS | 0.001 | 0.001 | 0.002 | 0.005 | 0.007$^{B}$ | 0.004$^{B}$ | | |
| | SB | 0.001$^{b}$ | <0.001$^{b}$ | <0.001$^{b}$ | 0.002$^{b}$ | 0.185$^{Aa}$ | 0.180$^{Aa}$ | | |
| Eubacteriaceae | | | | | | | | | |
| | WS | 0.015 | 0.021 | 0.015$^{A}$ | 0.014$^{A}$ | 0.015$^{A}$ | 0.013 | **<.001** | **<.001** |
| | WB | 0.020 | 0.014 | 0.012$^{A}$ | 0.017$^{A}$ | 0.018$^{A}$ | 0.014 | | |
| | SS | 0.016$^{a}$ | 0.018$^{a}$ | 0.017$^{Aa}$ | 0.006$^{Bb}$ | 0.006$^{Bb}$ | 0.013$^{a}$ | | |
| | SB | 0.017$^{a}$ | 0.014$^{a}$ | 0.003$^{Bb}$ | 0.004$^{Bb}$ | 0.003$^{Bb}$ | 0.010$^{ab}$ | | |
| Lachnospiraceae | | | | | | | | | |
| | WS | 0.097 | 0.124 | 0.090$^{A}$ | 0.112$^{A}$ | 0.097$^{A}$ | 0.111$^{A}$ | **.017** | **<.001** |
| | WB | 0.117 | 0.100 | 0.085$^{A}$ | 0.109$^{A}$ | 0.088$^{A}$ | 0.134$^{A}$ | | |
| | SS | 0.103 | 0.100 | 0.133$^{A}$ | 0.146$^{A}$ | 0.076$^{A}$ | 0.085$^{A}$ | | |
| | SB | 0.111$^{a}$ | 0.099$^{a}$ | 0.019$^{Bb}$ | 0.006$^{Bb}$ | 0.032$^{Bb}$ | 0.026$^{Bb}$ | | |
| Lactobacillaceae | | | | | | | | | |
| | WS | <0.001 | 0.001 | <0.001 | <0.001$^{B}$ | 0.002$^{B}$ | 0.001 | **<.001** | **<.001** |
| | WB | 0.005 | 0.004 | 0.003 | 0.007$^{B}$ | 0.036$^{B}$ | 0.015 | | |
| | SS | 0.004$^{b}$ | 0.006$^{b}$ | 0.004$^{b}$ | 0.073$^{Bb}$ | 0.233$^{Aa}$ | 0.046$^{b}$ | | |
| | SB | 0.002$^{b}$ | 0.001$^{b}$ | 0.050$^{b}$ | 0.254$^{Aa}$ | 0.096$^{Aa}$ | 0.055$^{b}$ | | |
| Methanobacteriaceae | | | | | | | | | |
| | WS | 0.052 | 0.037 | 0.057$^{A}$ | 0.019 | 0.016$^{A}$ | 0.013$^{A}$ | **.002** | **.003** |
| | WB | 0.046 | 0.017 | 0.027$^{A}$ | 0.037 | 0.019$^{A}$ | 0.037$^{A}$ | | |
| | SS | 0.031 | 0.012 | 0.013$^{A}$ | 0.009 | 0.012$^{A}$ | 0.049$^{A}$ | | |
| | SB | 0.031$^{a}$ | 0.025$^{a}$ | 0.130$^{Bc}$ | 0.022$^{a}$ | 0.002$^{Bb}$ | <0.001$^{Bb}$ | | |

| | | | | | | | | | |
|---|---|---|---|---|---|---|---|---|---|
| Prevotellaceae | | | | | | | | | |
| | WS | 0.017 | 0.015 | 0.012 | 0.018 | $0.014^{B}$ | $0.025^{B}$ | **.038** | **.004** |
| | WB | 0.017 | 0.025 | 0.018 | 0.015 | $0.031^{B}$ | $0.026^{B}$ | | |
| | SS | 0.015 | 0.020 | 0.024 | 0.062 | $0.021^{B}$ | $0.017^{B}$ | | |
| | SB | $0.018^{b}$ | $0.029^{b}$ | $0.002^{b}$ | $<0.001^{b}$ | $0.175^{Aa}$ | $0.128^{Aa}$ | | |
| Ruminococcaceae | | | | | | | | | |
| | WS | 0.109 | 0.133 | $0.115^{A}$ | $0.122^{A}$ | $0.105^{A}$ | $0.112^{A}$ | **<.001** | **<.001** |
| | WB | 0.132 | 0.119 | $0.126^{A}$ | $0.130^{A}$ | $0.083^{A}$ | $0.118^{A}$ | | |
| | SS | 0.122 | 0.140 | $0.129^{A}$ | $0.100^{A}$ | $0.071^{A}$ | $0.125^{A}$ | | |
| | SB | $0.112^{a}$ | $0.099^{b}$ | $0.014^{Bb}$ | $0.002^{Bb}$ | $0.001^{Bb}$ | $0.007^{Bb}$ | | |
| Spirochaetaceae | | | | | | | | | |
| | WS | 0.029 | 0.030 | $0.035^{A}$ | $0.028^{A}$ | $0.027^{A}$ | $0.040^{A}$ | **.002** | **<.001** |
| | WB | 0.022 | 0.028 | $0.024^{A}$ | $0.018^{A}$ | $0.039^{A}$ | $0.030^{A}$ | | |
| | SS | 0.026 | 0.024 | $0.018^{A}$ | $0.011^{A}$ | $0.017^{A}$ | $0.011^{A}$ | | |
| | SB | $0.017^{a}$ | $0.047^{a}$ | $0.006^{Bb}$ | $0.002^{Bb}$ | $0.001^{Bb}$ | $<0.001^{Bb}$ | | |
| Streptococcaceae | | | | | | | | | |
| | WS | 0.171 | 0.120 | $0.097^{B}$ | $0.111^{B}$ | 0.113 | 0.066 | **<.001** | **<.001** |
| | WB | 0.014 | 0.009 | $0.012^{B}$ | $0.031^{B}$ | 0.088 | 0.015 | | |
| | SS | 0.019 | 0.022 | $0.038^{B}$ | $0.069^{B}$ | 0.073 | 0.030 | | |
| | SB | $0.081^{b}$ | $0.074^{b}$ | $0.633^{Aa}$ | $0.625^{Aa}$ | $0.187^{b}$ | $0.226^{b}$ | | |

WS = water-saline; WB = water-buffer; SS = starch-saline; SB = starch-buffer. T*T = interaction treatment/time. T0 – before administration (starch overload and/or buffer); T8 – 8 hours administration; T12 – 12 hours after administration; T24 – 24 hours after administration; T48 – 48 hours after administration; T72 – 72 hours after administration. The means are represented: different lowercase letters on the same line, and different uppercase letters in the same column differed statistically (*$p<0.05$*; Tukey’s test).

Table S4. Variation in the relative abundance of the bacterial genera in the feces of horses 72 hours following treatments.

| Genus | Treatments | Time | | | | | | Time | T*T |
|---|---|---|---|---|---|---|---|---|---|
| | | T0 | T8 | T12 | T24 | T48 | T72 | P value | |
| Bifidobacterium | | | | | | | | | |
| | WS | <0.001 | <0.001 | <0.001 | <0.001 | <0.001$^{B}$ | <0.001$^{B}$ | **.016** | **.021** |
| | WB | <0.001 | <0.001 | <0.001 | <0.001 | <0.001$^{B}$ | <0.001$^{B}$ | | |
| | SS | <0.001 | <0.001 | <0.001 | 0.001 | 0.050$^{B}$ | 0.015$^{B}$ | | |
| | SB | <0.001$^{b}$ | <0.001$^{b}$ | <0.001$^{b}$ | 0.030$^{b}$ | 0.146$^{Aa}$ | 0.194$^{Aa}$ | | |
| Clostridium_III | | | | | | | | | |
| | WS | 0.026 | 0.027 | 0.024$^{A}$ | 0.022$^{A}$ | 0.022$^{A}$ | 0.024$^{A}$ | **<.001** | **<.001** |
| | WB | 0.025 | 0.020 | 0.022$^{A}$ | 0.023$^{A}$ | 0.014$^{A}$ | 0.023$^{A}$ | | |
| | SS | 0.025$^{a}$ | 0.028$^{a}$ | 0.023$^{Aa}$ | 0.012$^{Ab}$ | 0.011$^{Ab}$ | 0.019$^{Aa}$ | | |
| | SB | 0.026$^{a}$ | 0.022$^{a}$ | 0.003$^{Bb}$ | <0.001$^{Bb}$ | <0.001$^{Bb}$ | <0.001$^{Bb}$ | | |
| Clostridium_XIVa | | | | | | | | | |
| | WS | 0.051 | 0.070 | 0.054$^{A}$ | 0.061$^{A}$ | 0.056$^{A}$ | 0.072$^{A}$ | **<.001** | **<.001** |
| | WB | 0.067 | 0.065 | 0.053$^{A}$ | 0.058$^{A}$ | 0.055$^{A}$ | 0.077$^{A}$ | | |
| | SS | 0.047 | 0.056 | 0.061$^{A}$ | 0.068$^{A}$ | 0.032$^{A}$ | 0.038$^{A}$ | | |
| | SB | 0.061$^{a}$ | 0.065$^{a}$ | 0.008$^{Bb}$ | 0.002$^{Bb}$ | 0.002$^{Bb}$ | 0.006$^{Bb}$ | | |
| Erysipelotrichaceae* | | | | | | | | | |
| | WS | <0.001 | <0.001 | <0.001 | <0.001 | <0.001$^{B}$ | <0.001$^{B}$ | **.002** | **<.001** |
| | WB | 0.001 | 0.001 | 0.001 | 0.002 | <0.001$^{B}$ | <0.001$^{B}$ | | |
| | SS | 0.001 | <0.001 | 0.001 | 0.002 | 0.005$^{B}$ | 0.003$^{B}$ | | |
| | SB | 0.001$^{b}$ | <0.001$^{b}$ | <0.001$^{b}$ | 0.002$^{b}$ | 0.183$^{Aa}$ | 0.173$^{Aa}$ | | |
| Eubacterium | | | | | | | | | |
| | WS | 0.015 | 0.020 | 0.015$^{A}$ | 0.014$^{A}$ | 0.015$^{A}$ | 0.013 | **<.001** | **<.001** |
| | WB | 0.019 | 0.014 | 0.011$^{A}$ | 0.017$^{A}$ | 0.018$^{A}$ | 0.014 | | |
| | SS | 0.016$^{a}$ | 0.018$^{a}$ | 0.016$^{Aa}$ | 0.006$^{Bb}$ | 0.006$^{Bb}$ | 0.013$^{a}$ | | |
| | SB | 0.017$^{a}$ | 0.014$^{a}$ | 0.003$^{Bb}$ | 0.003$^{Bb}$ | 0.003$^{Bb}$ | 0.010$^{a}$ | | |
| Lachnospiraceae* | | | | | | | | | |
| | WS | 0.022 | 0.030 | 0.020$^{A}$ | 0.021$^{A}$ | 0.024$^{A}$ | 0.024$^{A}$ | **.027** | **.002** |
| | WB | 0.025 | 0.020 | 0.019$^{A}$ | 0.028$^{A}$ | 0.017$^{A}$ | 0.033$^{A}$ | | |
| | SS | 0.028 | 0.023 | 0.034$^{A}$ | 0.035$^{A}$ | 0.018$^{A}$ | 0.027$^{A}$ | | |
| | SB | 0.027$^{a}$ | 0.019$^{a}$ | 0.006$^{Bb}$ | 0.002$^{Bb}$ | 0.003$^{Bb}$ | 0.008$^{Bb}$ | | |
| Lactobacillus | | | | | | | | | |
| | WS | <0.001 | 0.001 | 0.001 | 0.001$^{B}$ | 0.002$^{B}$ | 0.001 | **<.001** | **.001** |
| | WB | 0.005 | 0.004 | 0.003 | 0.007$^{B}$ | 0.036$^{B}$ | 0.015 | | |
| | SS | 0.004$^{b}$ | 0.006$^{b}$ | 0.004$^{b}$ | 0.073$^{Bab}$ | 0.233$^{Aa}$ | 0.046$^{b}$ | | |
| | SB | 0.002$^{b}$ | 0.001$^{b}$ | 0.050$^{b}$ | 0.254$^{Aa}$ | 0.095$^{Aab}$ | 0.055$^{b}$ | | |
| Methanobrevibacter | | | | | | | | | |
| | WS | 0.052 | 0.036 | 0.057$^{A}$ | 0.019 | 0.016$^{A}$ | 0.013$^{A}$ | **.002** | **.003** |
| | WB | 0.046 | 0.017 | 0.027$^{A}$ | 0.037 | 0.009$^{A}$ | 0.037$^{A}$ | | |
| | SS | 0.031 | 0.012 | 0.013$^{A}$ | 0.009 | 0.012$^{A}$ | 0.049$^{A}$ | | |
| | SB | 0.031$^{a}$ | 0.025$^{a}$ | 0.130$^{Bc}$ | 0.022$^{a}$ | 0.002$^{Bb}$ | <0.001$^{Bb}$ | | |
| Prevotella | | | | | | | | | |
| | WS | 0.015 | 0.013 | 0.010 | 0.016 | 0.013$^{B}$ | 0.023$^{B}$ | **.031** | **.003** |
| | WB | 0.015 | 0.023 | 0.016 | 0.014 | 0.031$^{B}$ | 0.020$^{B}$ | | |
| | SS | 0.015 | 0.019 | 0.023 | 0.062 | 0.021$^{B}$ | 0.016$^{B}$ | | |
| | SB | 0.014$^{b}$ | 0.021$^{b}$ | 0.002$^{b}$ | <0.001$^{b}$ | 0.175$^{Aa}$ | 0.128$^{Aa}$ | | |
| Ruminococcaceae* | | | | | | | | | |
| | WS | 0.037 | 0.051 | 0.047$^{A}$ | 0.049$^{A}$ | 0.046$^{A}$ | 0.045$^{A}$ | **<.001** | **.001** |
| | WB | 0.062 | 0.056 | 0.061$^{A}$ | 0.069$^{A}$ | 0.037$^{A}$ | 0.057$^{A}$ | | |
| | SS | 0.059 | 0.066 | 0.061$^{A}$ | 0.062$^{A}$ | 0.037$^{A}$ | 0.061$^{A}$ | | |
| | SB | 0.049$^{a}$ | 0.047$^{a}$ | 0.008$^{Bb}$ | 0.001$^{Bb}$ | 0.001$^{Bb}$ | 0.004$^{Bb}$ | | |
| Ruminococcus | | | | | | | | | |
| | WS | 0.025 | 0.035 | 0.025$^{A}$ | 0.032$^{A}$ | 0.016$^{A}$ | 0.023$^{A}$ | **<.001** | **.050** |
| | WB | 0.015 | 0.017 | 0.014$^{A}$ | 0.009$^{A}$ | 0.011$^{A}$ | 0.012$^{A}$ | | |
| | SS | 0.012$^{a}$ | 0.015$^{a}$ | 0.016$^{Aa}$ | 0.003$^{Bb}$ | 0.003$^{Bb}$ | 0.005$^{Bb}$ | | |
| | SB | 0.014$^{a}$ | 0.013$^{a}$ | 0.001$^{Bb}$ | <0.001$^{Bb}$ | <0.001$^{Bb}$ | <0.001$^{Bb}$ | | |
| Streptococcus | | | | | | | | | |
| | WS | 0.171 | 0.120 | 0.099$^{B}$ | 0.110$^{B}$ | 0.099 | 0.066 | **<.001** | **.001** |
| | WB | 0.014 | 0.009 | 0.011$^{B}$ | 0.031$^{B}$ | 0.088 | 0.015 | | |
| | SS | 0.019 | 0.022 | 0.038$^{B}$ | 0.069$^{B}$ | 0.073 | 0.030 | | |
| | SB | 0.081$^{b}$ | 0.074$^{b}$ | 0.633$^{Aa}$ | 0.625$^{Aa}$ | 0.187$^{b}$ | 0.126$^{b}$ | | |
| Treponema | | | | | | | | | |
| | WS | 0.029 | 0.030 | 0.035 | 0.028$^{A}$ | 0.027$^{A}$ | 0.040$^{A}$ | **.002** | **<.001** |
| | WB | 0.022 | 0.028 | 0.024 | 0.018$^{A}$ | 0.039$^{A}$ | 0.030$^{A}$ | | |
| | SS | 0.026 | 0.024 | 0.018 | 0.011$^{A}$ | 0.017$^{A}$ | 0.011$^{A}$ | | |

| | | | | | | |
|---|---|---|---|---|---|---|
| SB | $0.017^{a}$ | $0.027^{a}$ | $0.016^{a}$ | $0.002^{Bb}$ | $0.001^{Bb}$ | $<0.001^{Bb}$ |

WS = water-saline; WB = water-buffer; SS = starch-saline; SB = starch-buffer. T*T = interaction treatment/time T0 – before gastric administration of water or starch; T8 - 08h; T12 - 12h; T24 - 24h; T48 - 48h; T72 - 72h. The means are represented: different lowercase letters on the same line, and different uppercase letters in the same column differed statistically ($p<0.05$; Tukey's test). *Genus not identified belonging to Family mentioned.
