# Supplementary material for: Effects of Starch Overload and Cecal Buffering on Fecal Microbiota of Horses
Source: Animals (Basel). 2022 Dec 6;12(23):3435. doi: 10.3390/ani12233435 (PMC9737938; doi:10.3390/ani12233435)
Supplement: Supplementary file 1 [file animals-12-03435-s001.zip › Tables S5--S7.pdf]

Table S5. Pearson's product-moment correlation between the composition of bacterial phyla found in horses' fecal microbiota and clinical alterations related to acute laminitis in animals from starch-saline and starch-buffer treatments.

**Observed Parameters**	**Euryarcheota**		**Actinobacteria**		**Bacteroidetes**		**Firmicutes**		**Proteobacteria**		**Spirochaetes**	
**SS**												
	r	p	r	p	r	p	r	p	r	p	r	p
**HR**	0.2	.248	0.0	.639	0.1	.583	0.0	.977	-0.1	.478	0.0	.800
**T°C**	-0.1	.739	0.5	**.011**	-0.1	.501	0.1	.716	-0.2	.333	0.0	.830
**Diarrhea**	-0.3	.139	0.4	**.017**	0.3	.157	0.5	**.004**	0.6	**.001**	-0.3	.080
**Inappetence**	-0.2	.461	0.0	.991	-0.1	.513	0.5	**.004**	0.6	**.002**	-0.1	.479
**Decubitus**	0.0		0.0		0.0		0.0		0.0		0.0	
**Dehydration**	-0.2	.231	0.0	.867	0.6	**.002**	0.2	.207	0.5	**.005**	-0.1	.581
**N° steps**	-0.2	.317	0.5	.006	-0.1	.438	0.1	.585	0.3	.160	0.0	.852
**Obel Grade**	0.1	.672	0.1	.653	-0.1	.594	0.2	.267	0.3	.124	-0.1	.445
**SB**												
	**Euryarcheota**		**Actinobacteria**		**Bacteroidetes**		**Firmicutes**		**Proteobacteria**		**Spirochaetes**	
	r	p	r	p	r	p	r	p	r	p	r	p
**HR**	0.1	.735	0.6	**.002**	-0.1	.783	0.3	.073	-0.1	.777	-0.4	**.017**
**T°C**	0.5	**.010**	-0.3	.144	-0.1	.464	0.3	.069	-0.3	.170	-0.2	.337
**Diarrhea**	-0.1	.682	0.1	.550	-0.4	.057	0.7	**.001**	-0.2	.406	-0.3	.105
**Inappetence**	0.0	.912	0.4	.052	0.1	.710	0.3	.173	0.3	.154	-0.2	.217
**Decubitus**	0.0	.847	0.4	**.019**	-0.1	.496	0.3	.158	-0.1	.701	0.1	.452
**Dehydration**	-0.2	.333	0.5	**.003**	-0.4	**.041**	0.5	**.003**	-0.2	.387	-0.3	.070
**N° steps**	-0.2	.391	0.3	.071	0.3	**.060**	0.2	.428	0.3	.115	-0.3	.094
**Obel Grade**	-0.3	.168	0.7	**.001**	0.1	.757	0.0	.934	0.0	.989	-0.3	.148

SS = starch-saline; SB = starch-buffer; HR = heart rate (beats/minute); T°C = rectal temperature; N° steps = number of steps; r = correlation (negative - r<0.1 [beige]; weak - 0.1<r<0.3 [light blue]; moderate - 0.4<r<0.6 [blue]; strong - 0.7<r<1.0 [dark blue]).

Table S6. Pearson's product-moment correlation between the composition of bacterial families found in horses' fecal microbiota and clinical alterations related to acute laminitis in animals from starch-saline and starch-buffer treatments.

		Observed Parameters							
		HR	T°C	Diarrhea	Inappetence	Decubitus	Dehydration	N° steps	Obel Grade
**SS**									
**Ruminococcacea**	**r**	0.5	0.1	-0.5	-0.4	0.0	-0.4	-0.3	-0.1
	**p**	**.010**	.617	**.010**	.056		**.049**	.088	.604
**Lachnospiraceae**	**r**	0.2	-0.2	-0.3	-0.3	0.0	-0.1	-0.4	-0.3
	**p**	.424	.348	.187	.102		.638	**.036**	.090
**Lactobacillaceae**	**r**	-0.3	0.0	0.5	0.6	0.0	0.2	0.4	0.3
	**p**	.185	.910	**.005**	**<.001**		.317	**.049**	.135
**Prevotellaceae**	**r**	0.0	-0.1	0.2	-0.1	0.0	0.5	-0.2	-0.2
	**p**	.643	.516	.293	.476		**.010**	.339	.313
**Methanobacteriaceae**	**r**	0.2	0.0	-0.3	-0.1	0.0	-0.2	-0.2	0.1
	**p**	.269	.839	.178	.503		.283	.336	.590
**Spirochaetaceae**	**r**	0.0	0.0	-0.4	-0.2	0.0	-0.1	-0.2	-0.1
	**p**	.810	.868	**.043**	.359		.519	.358	.445
**Streptococcaceae**	**r**	-0.1	0.1	0.6	0.3	0.0	0.5	0.0	0.1
	**p**	.567	.595	**<.001**	.115		**.004**	.663	.604
**Bifidobacteriaceae**	**r**	-0.1	0.4	0.5	0.0	0.0	0.0	0.6	0.1
	**p**	.615	**.015**	**.010**	.821		.917	**.002**	.547
**Erysipelotrichaceae**	**r**	0.0	0.4	0.4	0.0	0.0	0.0	0.4	0.0
	**p**	.919	**.016**	**.045**	.673		.886	**.035**	.824
**Eubacteriaceae**	**r**	0.0	0.0	-0.5	-0.3	0.0	-0.4	-0.3	-0.3
	**p**	.690	.742	**.006**	.089		**.045**	.072	.086
**SB**									
**Ruminococcacea**	**r**	-0.6	-0.4	-0.7	-0.4	0.0	-0.6	-0.3	-0.2
	**p**	**<.001**	**.019**	**<.001**	**.024**	.957	**<.001**	.116	.265
**Lachnospiraceae**	**r**	-0.6	-0.3	-0.6	-0.4	0.0	-0.7	-0.4	-0.4
	**p**	**<.001**	.110	**<.001**	**.046**	.899	**<.001**	**.035**	.051
**Lactobacillaceae**	**r**	0.2	0.0	0.6	0.3	-0.2	0.6	0.0	0.1
	**p**	.335	.809	**.001**	.090	.385	**<.001**	.886	.779

**Prevotellaceae**	**r**	-0.1	-0.1	-0.3	0.0	-0.1	-0.4	0.3	0.0
	**p**	.542	.535	.058	.791	.470	**.033**	.155	>.999
**Methanobacteriaceae**	**r**	0.1	0.5	-0.1	0.0	0.0	-0.2	-0.2	-0.3
	**p**	.669	**.008**	.643	.854	.883	.344	.408	.183
**Spirochaetaceae**	**r**	-0.4	-0.1	-0.3	-0.2	0.2	-0.3	-0.3	-0.3
	**p**	**.041**	.588	.169	.310	.389	.114	.155	.168
**Streptococcaceae**	**r**	0.2	0.4	0.5	0.2	-0.2	0.3	-0.1	-0.2
	**p**	.314	**.019**	**.003**	.368	.268	.124	.605	.284
**Bifidobacteriaceae**	**r**	0.5	-0.3	0.1	0.3	0.4	0.5	0.3	0.7
	**p**	**.002**	.148	.576	.059	**.020**	**.003**	.098	**<.001**
**Erysipelotrichaceae**	**r**	0.4	0.0	0.1	0.1	0.1	0.2	0.8	0.5
	**p**	**.014**	.963	.612	.468	.688	.294	**<.001**	**.002**
**Eubacteriaceae**	**r**	-0.6	-0.4	-0.7	-0.4	0.0	-0.6	-0.3	-0.2
	**p**	**<.001**	**.019**	**<.001**	**.024**	.957	**<.001**	.116	.265

SS = starch-saline; SB = starch-buffer; HR = heart rate (beats/minute); T°C = rectal temperature; N° steps = number of steps; r = correlation (negative - r<0.1 [beige]; weak - 0.1<r<0.3 [light blue]; moderate - 0.4<r<0.6 [blue]; strong - 0.7<r<1.0 [dark blue]).

Table S7. Pearson's product-moment correlation between the composition of bacterial genera found in horses' fecal microbiota and clinical alterations related to acute laminitis in animals from starch-saline and starch-buffer treatments.

		Observed Parameters							
		HR	T°C	Diarrhea	Inappetence	Decubitus	Dehydration	N° steps	Obel Grade
**SS**									
**Clostridium_XIVa**	r	0.2	-0.2	-0.3	-0.3	0.0	-0.1	-0.3	-0.4
	p	.421	.387	.107	.077		.539	.091	.059
**Ruminococcaceae***	r	0.4	0.0	-0.3	-0.3	0.0	-0.3	-0.3	-0.1
	p	**.032**	.866	.067	.114		.098	.083	.608
**Lactobacillus**	r	-0.3	0.0	0.5	0.6	0.0	0.2	0.4	0.3
	p	.187	.930	**.005**	**<.001**		.315	.053	.138
**Methanobrevibacter**	r	0.2	0.0	-0.3	-0.1	0.0	-0.2	-0.2	0.1
	p	.262	.805	.182	.502		.295	.329	.599
**Prevotella**	r	0.0	-0.2	0.3	-0.1	0.0	0.5	-0.2	-0.2
	p	.828	.400	.176	.575		**.004**	.410	.406
**Treponema**	r	0.0	0.0	-0.3	-0.1	0.0	-0.1	0.0	-0.1
	p	.800	.830	.080	.479		.581	.852	.445
**Lachnospiraceae***	r	0.2	0.0	-0.2	-0.3	0.0	-0.2	-0.4	-0.1
	p	.266	.741	.192	.157		.403	**.038**	.451
**Streptococcus**	r	-0.1	0.1	0.6	0.3	0.0	0.5	0.1	0.1
	p	.539	.566	**<.001**	.125		**.006**	.609	.626
**Bifidobacterium**	r	-0.1	0.5	0.5	0.0	0.0	0.0	0.5	0.1
	p	.666	**.013**	**.012**	.869		.954	**.002**	.509
**Clostridium_III**	r	0.2	0.0	-0.5	-0.3	0.0	-0.4	-0.4	-0.3
	p	.409	.912	**.005**	.070		**.034**	**.043**	.093
**Erysipelotrichaceae***	r	0.0	0.4	0.4	-0.1	0.0	0.0	0.4	0.1
	p	.875	.053	**.024**	.750		.819	**.031**	.529
**Ruminococcus**	r	0.0	0.0	-0.3	-0.2	0.0	-0.2	-0.1	-0.2
	p	.900	.995	.080	.298		.322	.774	.202
**Eubacterium**	r	0.1	-0.1	-0.5	-0.3	0.0	-0.4	-0.3	-0.3
	p	.707	.767	**.006**	.113		**.034**	.103	.127
**SB**									
**Clostridium_XIVa**	r	-0.6	-0.3	-0.5	-0.4	0.0	-0.5	-0.6	-0.3
	p	**<.001**	.124	**.006**	**.020**	.860	**.002**	**.012**	.075
**Ruminococcaceae***	r	-0.6	-0.2	-0.5	-0.5	0.0	-0.5	-0.5	-0.3
	p	**<.001**	.253	**.008**	**.005**	.853	**.006**	**.010**	.068
**Lactobacillus**	r	0.2	0.0	0.6	0.3	-0.2	0.6	0.0	0.0
	p	.351	.806	**.001**	.102	.365	**<.001**	.864	.793
**Methanobrevibacter**	r	0.1	0.5	-0.1	0.0	0.0	-0.2	-0.2	-0.2
	p	.655	**.007**	.672	.852	.866	.352	.428	.193
**Prevotella**	r	-0.1	-0.1	-0.3	0.1	-0.1	-0.4	0.3	0.0
	p	.688	.575	.085	.703	.441	**.050**	.120	.895
**Treponema**	r	-0.4	-0.2	-0.3	-0.2	0.1	-0.3	-0.3	-0.3
	p	**.017**	.337	.105	.217	.452	.070	.094	.148
**Lachnospiraceae***	r	-0.6	-0.2	-0.6	-0.6	0.0	-0.6	-0.5	-0.3
	p	**<.001**	.247	**.001**	**<.001**	.909	**<.001**	**.009**	.073

**Streptococcus**	**r**	0.2	0.4	0.5	0.2	-0.2	0.3	-0.1	-0.2
	**p**	.329	**.021**	**.003**	.358	.264	.120	.587	.269
**Bifidobacterium**	**r**	0.5	-0.3	0.1	0.4	0.4	0.5	0.3	0.7
	**p**	**.002**	.152	.558	.056	**.019**	**.003**	.095	**<.001**
**Clostridium_III**	**r**	-0.6	-0.2	-0.5	-0.5	0.0	-0.5	-0.5	-0.3
	**p**	**<.001**	.213	**.009**	**.006**	.952	**.006**	**.006**	.058
**Erysipelotrichaceae***	**r**	0.4	0.0	0.1	0.1	0.1	0.2	0.8	0.5
	**p**	**.013**	.944	.599	.459	.710	.289	**<.001**	**.002**
**Ruminococcus**	**r**	-0.6	-0.3	-0.5	-0.5	-0.1	-0.5	-0.5	-0.3
	**p**	**.001**	.180	**.009**	**.009**	.447	**.004**	**.013**	.083
**Eubacterium**	**r**	-0.5	-0.4	-0.6	-0.4	0.0	-0.6	-0.2	-0.2
	**p**	**.004**	**.033**	**<.001**	**.036**	.831	**<.001**	.200	.366

SS = starch-saline; SB = starch-buffer; HR = heart rate (beats/minute); T°C = rectal temperature; N° steps = number of steps; r = correlation (negative - r<0.1 [beige]; weak - 0.1<r<0.3 [light blue]; moderate - 0.4<r<0.6 [blue]; strong - 0.7<r<1.0 [dark blue]).
